# The Impact of Confluence on Bone Marrow Mesenchymal Stem (BMMSC) Proliferation and Osteogenic Differentiation

**Published:** 2017-04-01

**Authors:** Faten A.M. Abo-Aziza, Zaki A.A

**Affiliations:** 1 Department of Parasitology and Animal Diseases, Veterinary Research Division, National Research Center, Giza, Egypt; 2 Department of Physiology, College of Veterinary Medicine, Cairo University, Giza, Egypt

**Keywords:** BMMSCS, Confluence, Osteogenic differentiation, Proliferation

## Abstract

**Background: **In the field of cellular therapy, the impact of confluence degreeonharvesting or differentiation of BMMSCs and the effect of cell-to-cell contact remain controversial. Therefore, the effect of confluence on properties of BMMSCs was studied and efficiency of confluence-associated osteogenic differentiation was identified.

**Materials and** **Methods:** The impact of 20, 50, 70, 80 and 100% confluences on proliferation properties of BMMSCs, expression of ERK and p-ERK proteins and glucose consumption rate was studied. Efficiency of confluence-associated osteogenic differentiation was identified by determining calcium deposition, Alizarin Red staining, ALP activity and expression of osteopontin and osteocalcin genes.

**Results:** There was a correlation between confluence % and BMMSCs density. Viability was declined at the lower and higher confluences. The highest CFU-F, Brd-U uptake and population doubling were obtained at 80% confluence. ERK band intensity in 100% confluent BMMSCs was lower compared to other confluences. Bands of p-ERK were highly detectable in 70% and 80% confluences. Glucose consumption rate of 70% and 80% confluences in the last days were higher than 20% and 100% confluences. Although higher osteogenic differentiation was estimated at 80% confluence using calcium deposition, Alizarin Red staining and ALP activity, it was also extended at 100% confluence Osteopontin gene was expressed among all confluences including 100% confluence, while osteocalcin gene was expressed highly in 70% confluent cells.

**Conclusion:** We concluded that the optimum seeding density for maximal expansion and harvesting purposes is 80% confluence and for osteogenic differentiation up to 100% confluence is also acceptable.

## Introduction

 The interest in both the biology and potential therapeutic applications of adult bone marrow stem cells still exists.^1^ Bone marrow mesenchymal stem cells (BMMSCs) are adult stem cells which have the capability to give rise to a variety of cells in the laboratory, including skeletal tissues, fat and muscle cells.^2^ Many studies aimed to culture BMMSCs for a long period of time keeping their differentiation capacity in appropriate quantities for clinical applications, to be good candidates for tissue repair.^3^

In clinical purposes, extensive expansion of isolated BMMSCs in vitro is required to obtain adequate numbers of cells. However, most expansion protocols involved adherent culture on plastic surfaces and serial passage. Many variables including cell confluence were considered in optimizing the expansion of BMMSCs. Cell confluence is a critical factor because the degree of confluence might affect the biological properties of BMMSCs. Generally, BMMSCs are sub-cultured or harvested when they reach a specified degree of confluence, but until now there is no standard concerning optimal confluence.^4^ For example, the using of different criteria to describe the conditions when the cells need to be sub-cultured, such as 50% to 60% confluence,^5^^,^^6^ 70% to 90% confluence,^7^ sub-confluent (70% to 80%),^8^ 80% confluence,^9^^,^^10^ 80% to 90% confluence,^11^ 90% confluence,^12^ near confluence,^13^ approaching confluence^14^ or confluent.^15^^,^^16^

The outcomes of BMMSCs from clinical trials were varied because BMMSCs used to treat many conditions were cultured on variable levels of confluence. Therefore, it is important to better understand how confluence at the time of harvest affects the properties of BMMSCs.^17^ For cell expansion under these circumstances, developing a measure to determine BMMSCs confluence is in urgent need. To maintain consistent BMMSCs property, the optimal expansion of BMMSCs should be achieved by determining the best seeding density and timing of passage.^4^

Cells continuously receive clues from their environments by the activation of surface receptors and extra-cellular matrix. Inside the cells, there is a need to integrate diverse signaling pathways to trigger an appropriate biological response. One of these signal transduction molecules kinases is Extracellular Signal-Regulated Kinase (ERK).^18^ ERK is involved in the control of many fundamental cellular processes such as cell proliferation, survival, differentiation, motility and metabolism.^19^ ERK is activated by phosphorylation of both tyrosine and threonine residues.^20^^,^^21^ Activated ERK phosphorylates cytoplasmic, membranous and nuclear constituents played a pivotal role in the regulation of many cell functions.^22^ Control of cell differentiation by activated ERK included stem cell commitment to chondrogenesis or osteogenesis under cyclic compression,^23^ control of early osteogenesis by hydrostatic pressure,^24^ and stretch inhibition of adipogenesis.^25^ ERK plays an important role in the ECM-induced osteogenic differentiation process.^26^

It was known that bone healing is a highly complicated and regulated process. In certain situations, such as non-union fractures and diseases including osteoporosis and osteoarthritis, the normal bone repair and remodeling processes are often impaired.^27^Osteogenic progenitor cells have proved support in bone regeneration when they are locally transplanted into bone defects as well as poorly or non-healing fractures. Therefore, osteogenic MSC preparations have been used as new cell-based therapies needed to repair damaged skeletal tissues.^28^ For the detection of osteogenic differentiation, it was necessary to use alizarin red stain,^29^ alkaline phosphatase activity^30^ and/or determine some genes expression like osteocalcin and osteopontin.^31^ Therefore, the present work aim to explore the 20, 50, 70, 80 and 100% confluences on the proliferation capacity of BMMSCs and their osteogenic differentiation efficiency. 

## Materials and Methods

**Animals**

Three-month-old male rats weighted 175 – 200 gm were used. All experiments on animals were performed under the institutionally approved protocols for the use of animal research. 

**Isolation and Cultivation of Rat Bone Marrow Mesenchymal Stem Cells (BMMSCs)**

To isolate BMMSCs, femur bones from rats were isolated.^32^ Skin incision was made with a scalpel in the femoral epiphysis region and the muscle was sectioned up to the femoral bone. Femur bones from rats were isolated and flushed using PBS. Standardised osteotomy was created at the femur head as described previously^33^. Bone marrow was aspirated with a 5-ml syringe containing 5000 UI/ml of heparin. Two ml of bone marrow blood from femurs was used for mononuclear cell isolation by gradient centrifugation at 2000 rpm for 30 minutes at room temperature on same volume of Ficoll-Histopaque®-1077-Sigma. Mononuclear cell layer was then aspirated with a pipette, washed twice and suspended in alpha-minimum essential medium (-MEM, Invitrogen, Grand Island, NY, USA). After counting the cells with haemocytometer, the single suspension of bone marrow derived all nucleated cells were seeded at a density of 15 × 10^6^ into 100 mm culture dishes (Corning, USA) and incubated at 37°C and 5% CO2. After two days, the media was changed to remove non-adherent cells while the attached cells were maintained in -MEM supplemented with 20% fetal bovine serum (FBS, Equitech bio, Kerrville, TX, USA), 2 mM L-glutamine, 55 μM 2-mercaptoethanol, 100 U/ml penicillin, and 100 μg/ml streptomycin (Invitrogen).^28^The media was changed every 3 days thereafter until the colonies reached 20, 50, 70, 80 and 100% confluence as determined by microscope observation. The cells were harvested and designated as passage 1, and serial passage numbers were designated thereafter. The cultured BMMSCs of passage 3 were plated to the T-25 flask from identified confluence for further culture. When passage 3 BMMSCs reached the respective confluences, they were harvested (passage 4) and subjected for cell proliferation and differentiation as follows: 

**Cell Counting and Viability Assay**

Upon reaching 20, 50, 70, 80 and 100% confluence, BMMSCs from each confluence were washed with 10 mL of PBS twice, digested with 1 mL of Trypsin (Invitrogen, Life Technologies) and centrifuged at 2000 rpm for 5 min. The cell number was then counted and viability was assessed by use of the trypan blue exclusion method.^32^

**Colony Forming Unit-Fibroblastic (CFU-F) Assay**

One million cells of the BMMSCs upon reaching 20, 50, 70, 80 and 100% confluence were seeded on a T-25 cell culture flask (Nunc, Rochester, USA). The cultured cells were washed with PBS after 16 days and then stained with 1% toluidine blue solution in 2% paraformaldehyde. After microscopical examination, each cell cluster that contains more than 50 cells was considered as a colony.^28^The number of colonies was counted in 5 independent samples per each confluence group. 

**Cell Proliferation Assay**

The proliferation of each confluent BMMSCs was created using the bromodeoxyuridine (Brd-U) incorporation assay.^28^For each confluence, 1 × 10^4^ cells/well was seeded on two- well chamber slides (Nunc) for 2-3 days. The cultured wells were incubated with Brd-U solution (1:100) (Invitrogen) for 24 hours and then stained with a Brd-U staining kit (Invitrogen). Total and positive Brd-U cell numbers were counted in 5 images in each confluence. 

**Population Doubling Assay**

Population doubling (PD) and population doubling time (PDT) were calculated by use of the method described previously.^4^A total of 0.25 × 10^6^cells of BMMSCs from each confluence were seeded on 60 mm culture dishes. The cells were passaged upon reaching 20, 50, 70, 80 and 100% confluence. The number of BMMSCs at every passage was counted and the PD in each passage was calculated using equation log2 (harvested cells number/ plated cells number). The final PD for each confluence was determined by successive addition of total numbers in each passage until the cell division was ceased. The population doubling time (PDT) for each confluence was monitored in passage 5 -6 and in passage 11 - 12, respectively. 

**Western Blotting Analysis**

Protein was extracted from cells as previously described.^34^The adhered cells were washed with Dulbecco’s phosphate-buffered saline and dislodged by cell scraper. Cells were collected into tubes and centrifuged at 1500 RPM for 5 minutes. Then the cells were lysed with 180 μL of ice-cold cell lysis buffer and 20 μL fresh protease inhibitor cocktail (Haltä, Pierce) for 30 min on ice followed by 10 min centrifugation at 12,000 RPM, at 4°C to clarify the lysate. The supernatant (or protein mix) was transferred to a fresh tube and stored on ice or frozen at -20°C or -80°C. Protein concentrations were measured on a spectrophotometry. ERK and p-ERK were measured in 20, 50, 70, 80 and 100% confluent BMMSCs.^35^ Sample cells were prepared after being heated for 5 min at 95 °C in a sample buffer and equal aliquots were then run on prepared 10% SDS–polyacrylamide gel. Proteins were transferred to PVDF membrane filters and then blocked with 5% skim milk in TBST for 1 h. Afterwards, the membrane filters were incubated overnight at 4 °C with the primary antibodies against ERK and p-ERK (Cell Signaling Technology). The membranes were washed and the primary antibodies were detected by incubating with horseradish peroxidase-conjugated goat anti-rabbit or anti-mouse IgG (check it) (PharMingen). The filters were washed and developed using a chemiluminescence system (ECL, Amersham Biosciences, UK). β-actin on the same membrane was served as the loading control and the immunoreactive bands were then visualized. Band intensities of ERK and p-ERK were quantitatively analyzed by using IMAGEJ software and normalized using the corresponding control β-actin for each protein respectively 

**Glucose Consumption Test**

For monitoring BMMSCs markers of confluence, glucose was measured in BMMSCs culture supernatants, indicating the glucose consumption by BMMSCs isolated from different confluence as previously described^36^. One million cells of each confluence was cultured in 100mm culture dishes and supplemented with 10mg glucose/ml. The concentrations of glucose were estimated daily in the culture supernatant of BMMSCs using glucometer. The consumption was estimated as the quantity of glucose (mg/ml) consumed minus the concentration of cell culture at beginning. 

**In Vitro Osteogenic Differentiation Assay**

BMMSCs from each confluence were cultured under osteogenic culture conditions.^28^ BMMSCs were induced for 14 days in -MEM with 20% FBS, 2 mM L-glutamine, 55 M 2-ME, 100 M L-ascorbic acid 2-phosphate, 2 mM-glycerophosphate, 10 nM Dexamethasone, 100 U/ml penicillin and 100 g/ml streptomycin. The medium was changed every 3 days. Confirmation of osteogenesis was done by means of alizarin red staining (to highlight ECM calcification), calcium deposition assay, assessment of ALP activity, and expression of osteopontin as well as osteocalcin genes.

**Alizarin Red Staining**

After 4 weeks of culture in osteogenic condition, the cells were stained for extracellular mineralization as described earlier.^37^The cells in 60mm dishes were washed with PBS and fixed in 60% (v/v) isopropanol (Sigma-Aldrich). After 1 min, the cells were rehydrated with distilled water then alizarin red stain 1% (pH 4.1, Sigma-Aldrich) was added to each dish. The dishes were incubated at room temperature for 3 minandwashed4 times with distilled water then left to air dry. Finally, the % of total area alizarin stain was read in triplicate at 405 nm in 96-well format using opaque-walled, transparent- bottomed plates.^38^

**Calcium Assay**

The differentiation of osteoblasts was determined by calcium assay as described elsewhere.^39^Briefly, fixed quantities from each confluence were seeded into wells, washed twice with PBS and extracted off the wells in 0.5 N HCl. Accumulated calcium was removed from the cellular component by shaking for 5 h at 4◦C, followed by centrifugation at 2,000 g for 10 min. The supernatant was utilized for calcium determination using calcium colorimetric assay kit (Sigma-Aldrish). Total calcium was calculated from standard solutions prepared in parallel and expressed as mg/well after absorbance at 575 nm after measuring the absorbance at 575 nm.

**Alkaline Phosphatase (ALP) Activity**

The differentiation of osteoblasts was determined by ALP activity assay as described elsewhere.^40^Briefly, the cells were treated with 20 𝜇L/well of 0.1% Triton X-100 (Sigma-Aldrish) for 5 min at room temperature for cell lysis. 100 𝜇L/well of the ALP assay kit (Sigma-Aldrich) was then added to produce p-nitrophenol from the hydrolysis of p-nitrophenyl phosphate. The ALP activity of cell lysates was determined by measurement of absorbance at 405 nm caused by p-nitrophenol using a MRX Microplate Reader. 

**Reverse Transcriptase-Polymerase Chain Reaction (RT-PCR)**

RNA was isolated from 10 × 10^6^osteogenic BMMSCs differentiated from each confluence. The RNeasy mini kit (Qiagen, Valencia, CA) was used for total RNA isolation. RT-PCR was carried out with the OneStep RT- PCR Kit (Qiagen) and a 96-well thermal cycler using primers specific for osteopontin and osteocalcin listed in Table1. For each reaction,1 microgram of template RNA was used. The reverse transcription step was allowed to run for 30 min at 50°C, followed by PCR activation for 15 min at 95°C. Then 30 amplification cycles were run for denaturation, annealing and extension within 1 min at 94°C, 58°C and 72°C, respectively. The final extension was performed at 72°C for 10 min. The products were separated by gel electrophoresis using a 1% agarose gel. Bands were visualized using UV illumination of ethidium-bromide stained gels and were captured using Gel imaging system. 

**Statistical Analysis**

Data were analyzed with the Statistical Package for the Social Sciences version 19 (SPSS-19).

**Table 1:** Primer sequences used for RT-PCR

Gene name	Primer sequences	Product size(bp)
SSP1 (Osteopontin)	Forwards 5′ - AGACCCCAAAAGTAAGGAAGAAGA-3′ Reverse 5′- GACAACCGTGGGAAAACAAATAAG-3′	564
BGLAP (Osteocalcin)	Forwards 5′ - CGCAGCCACCGAGACACCAT-3′Reverse 5′- AGGGCAAGGGGAAGAGGAAAGAA-3′	400

## Results

 Microscopic examination of BMMSCs showed 20% confluence after 2-3 days in culture and 50% confluence after 2 weeks. Cells reached 70% confluence after 2-3 weeks, while they became 80% and 100% after 3-4 weeks respectively. 

**Figure 1:**
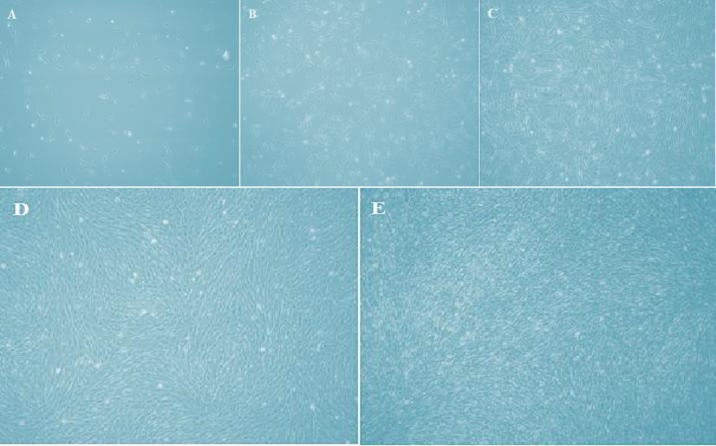
**Light Microscopy Images:** 20% confluent BMMSCs after 2-3 days in culture (A). 50% confluence after 2 weeks (B), after 2-3 weeks BMMSCs reached 70% confluence (C), after 3-4 weeks BMMSCs became 80% and 100% confluence and they lined next to each other (D, E). Magnification 10×.

BMMSCs among all confluences exhibited spindle-shaped morphology. BMMSCs appeared large and flattened in culture at 20, 50 and 70% confluence, but when BMMSCs reach 80% and 100% they became very confluent and lined next to each other (Figure 1).

**Cell Density**

**Figure 2:**
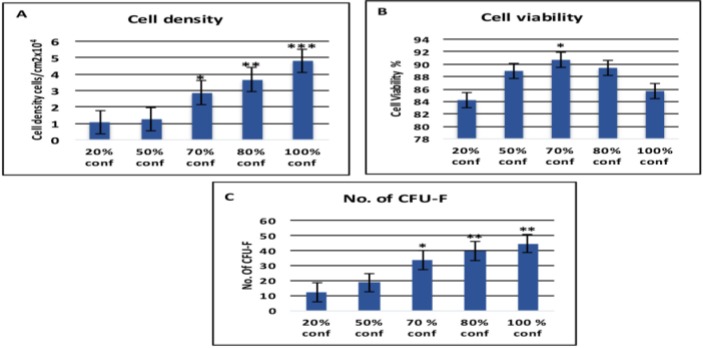
**(A) Cell Density:** The highest cell density appeared in 100% confluence (4.802x10000 cells/cm2) and the lowest density appeared in 20% confluent BMMSCs (1.089 x10000 cells/cm2). **(B) Cell viability:** The viability was less than 90% in all confluences except higher viability at 70% that recorded 90.71%. There was no significant difference in viability among 50%, 70% and 80%, while viability declined at both 20% and 100% confluences. **(C) Cell Forming Unit- Fibroblastic:** The number of plastic attached CFU-F from BMMSCs at 20, 50, 70, 80 and 100% confluences (1 × 106 cells). The highest number of CFU-F was in 80% confluence and then decreased with increasing the confluence to 100%. Error bars represent Mean ± SE. Asterisks *, ** and*** indicate significant at P<0.05, P<0.01, P<0.001 respectively compared with non-marked groups.

BMSCs were cultured to 20, 50%, 70, 80 and 100% confluence; the cell densities per cm^2^ culture area were calculated. it was found that higher BMMSC density correlated with increased confluence. It was also found that the highest cell density appeared when the cell achieved 100% confluence (4.802x10000 cells/cm^2^) and the lowest density appeared at 20% confluent BMMSCs (1.089 x10000 cells/cm^2^). However, cell density at 70% (2.887 x10000 cells/cm^2^) and 80% confluence (3.679 x10000 cells/cm^2^) was significantly lower than that at 100% confluent and higher than the others (Figure 2A).

**Cell Viability**

The viability was less than 90% in all confluences except at 70% that recorded 90.71%. There was no significant difference in viability among 50%, 70% and 80% confluences. BMMSCS viability decline at 20% (84.16%) and 100% (85.67%) confluences and no significant difference was observed between them (Figure 2B).

**CFU-F**

As the confluence of BMMSCs increases, the cells become highly adherent to the BMMSC-ECM and form CFU-F at a high extent until it reaches 80%, and then decreases with increasing the confluence to 100% (Figure 2C).

**Cell Proliferation Assay**

**Figure 3:**
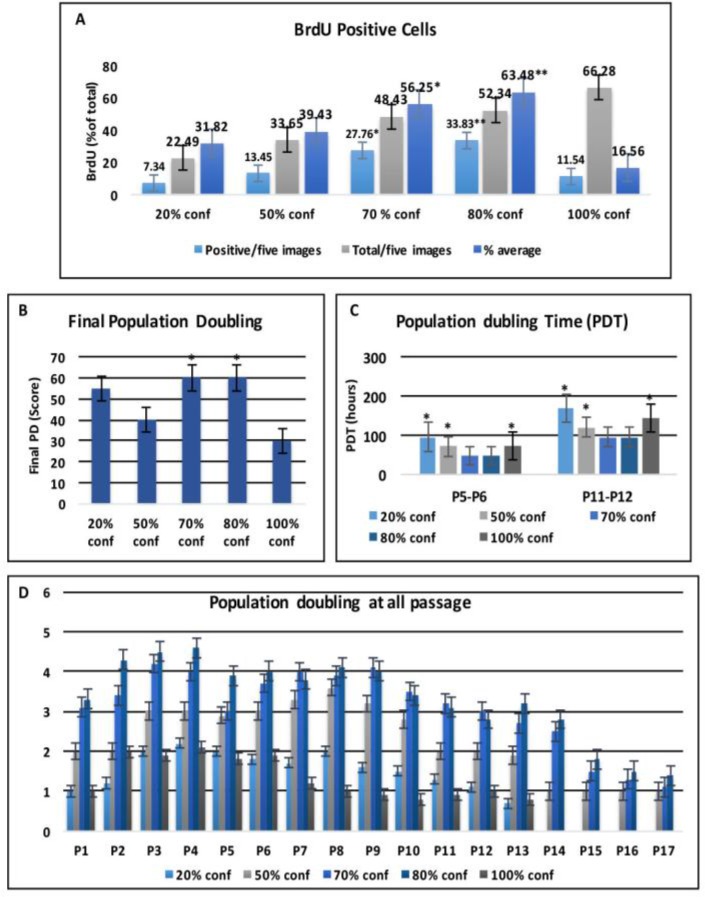
**Cell Proliferation Assay: (A)** Brd-U incorporation assay shows that the Brd-U uptake rate is significantly elevated as the confluence increased until it reached 80% confluence. **(B)** The proliferation of BMMSCs was determined by means of population doubling final score, **(C)** calculated population doubling time (PDT) in passage 5 to 6 and in passage 11 to 12 and **(D)** population doubling at all passages. Error bars represent Mean ± SE. Asterisks * and ** indicate significant at P<0.05, P<0.01 respectively compared with non-marked groups.

The proliferation rates of BMMSCs at different confluences were assessed by Brd-U incorporation for 24 hours. The Brd-U uptake rate was significantly elevated as the confluence increased until it reached the higher rate at 80% confluence .It was appeared that the percentage of positive cells is significantly higher (56.25% and 63.48%) in 70% and 80% confluent BMMSCs compared with 20%, 50%, and 100% confluence (31.82%, 39.43% and 16.56%), respectively (Figure 3A).

**Population Doubling**

The PD was calculated at every passage according to the equation log^2^ (number of harvested cells/number of seeded cells). Comparison of final PD score among the different confluences indicates that BMMSCs at 70% and 80% confluences exhibit a significant increase in PDs comparing to that of 50% and 100% confluences (Figure 3B). Population doubling time (PDT) was recorded in passage 5 to 6 and in passage 11 to 12, respectively. In both passages, the PDT of 70% and 80% confluent BMMSCs was slightly shorter than 50% confluent cells but much shorter than 20% and 100% confluent cells (Figure 3C). BMMSCs at 70% and 80% confluence showed elevated PD scores than other confluences in all passages. All confluent BMMSCs showed maximal expansion potential in passage 3-4. In most confluent BMMSCs, cell growth arrested in passage 12–13, whereas 70% and 80% confluent stopped in passage 14 - 16 (Figure 3D). 

**The Expression of ERK and P-ERK Proteins**

**Figure 4:**
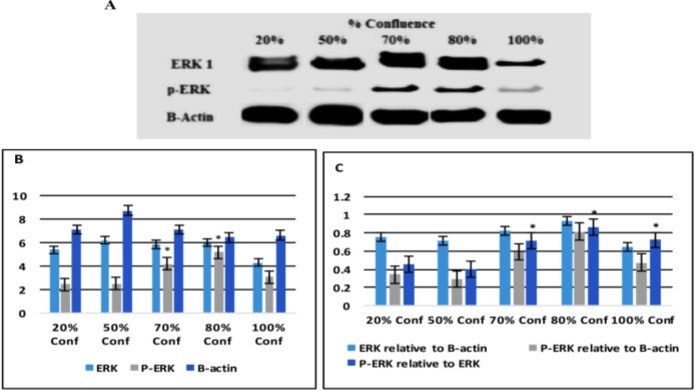
**Erk and P-ERK Protein Signaling Pathway:** Total ERK and P-ERK in 20, 50, 70, 80 and 100% confluent BMMSCs were detected by western blot. Immunoreactivity to B- actin was used as a loading control **(A)**.Densitometric measures of band intensity for ERK and P-ERK are shown by using IMAGEJ software **(B)**. Normalized band intensity of ERk and p-ERK relative to actin and P ERK related to ERK are shown by calculation **(C)**. Error bars represent Mean ± SE. Asterisks * indicate significant at P<0.05compared with non-marked groups.

The expression of ERK and p-ERK proteins was performed by Western blotting using anti ERK and p-ERK antibodies, respectively. As shown in Figure 4, bands of ERK were expressed in BMMSCs and detected by Western blot among all confluences. (Figure 4A). Bands of p-ERK were highly detectable at 70% and 80% confluence (Figure 4A). Densitometric measures of band intensities for ERK and p-ERK using IMAGEJ software and B-actin were used as controls. It was found that ERK band intensity of 100% confluent BMMSCs was low compared to other confluences. The intensities of remaining confluences bands were nearly the same. While the higher p-ERK band intensity was found in 70% and 80% confluent BMMSCs compared to other confluences (Figure 4B). Phosphorylation of ERK (p-ERK) in 80% confluent BMMSCs was much higher than 20% and 50% confluent cells but slightly higher than 70% and 100% confluent cells. Phosphorylation of ERK in 70% and 100% was higher than 20% and 50% confluences (Figure 4C). 

**Glucose Consumption Rate**

**Figure 5:**
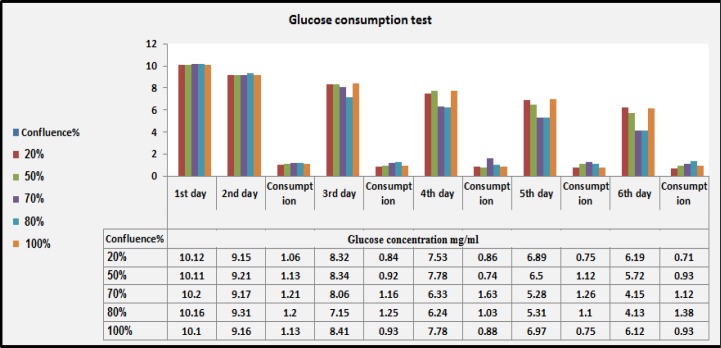
**Glucose Levels in BMMSC Supernatants:** Monitoring of glucose consumption in culture supernatant of BMMSCs using glucometer. The quantity of glucose (mg/ml) consumed minus the concentration of cell culture at beginning among different confluences.

For monitoring activity of BMSC confluences, we measured glucose of the BMSC culture supernatants. The concentrations of glucose were estimated daily in the culture supernatant of BMSC using glucometer. It was estimated as the quantity of glucose (mg/ml) consumed minus the concentration of cell culture at beginning. The glucose levels decreased daily despite small fluctuations after medium change on days 4, 5 and 6. All BMMSs in all confluences are regular in glucose consumption after day 2. However, on days 3, 4 and 6, glucose consumption of BMMSCs at 70% and 80% confluence was elevated than the others. On the day 5, the consumption rates of BMMSCs at confluence 50, 70, and 80% were higher than the lowest (20%) and highest (100%) confluences (Figure 5). 

**Osteogenic Differentiation**

**Figure 6:**
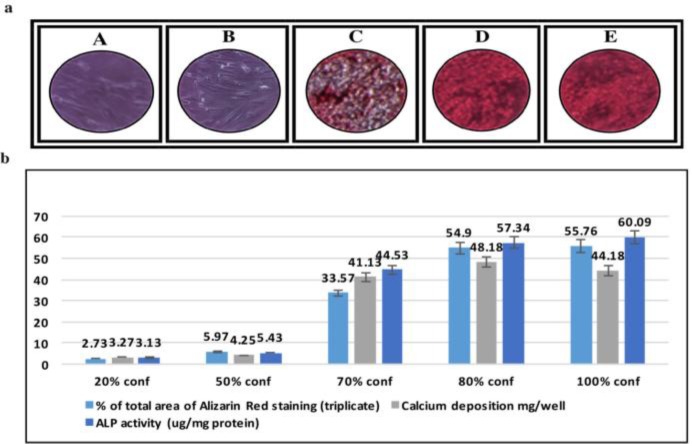
**In Vitro Osteogenic Differentiation:(a)** Alizarin Red staining showed that BMMSCs at 20% and 50 % confluence can not show in vitro osteogenic differentiation potential (A, B). 70, 80 and 100% confluences were similar in osteogenic differentiation in vitro (C, D, E). (b) % of total area stained with Alizarin Red dye, calcium deposition mg/well ALP activity (μg/mg protein) for osteogenic differentiated 20, 50, 70, 80 and 100% confluent BMMSCs.

To address whether confluence affects osteogenic differentiation of BMMSCs, % of total area of Alizarin Red staining, calcium deposition and the activity of alkaline phosphatase (ALP) were determined. Cells cultured in 20% and 50% did not cause a significant increase in % of total area of Alizarin Red staining as compared with the cells cultured at 70, 80 and 100% confluence (Figure 6 a&b). % of total area of Alizarin Red staining for both 80% and 100% confluent BMMSCs are significantly higher than that in the other confluences. Measurement of calcium deposition resulted in a more significant increase in OD of MScs cultured at 80% confluence than both 70% and 100% confluence. However, MSCs cultured in 100% confluence further increased the OD value of ALP activity However, MSCs cultured in 100% confluence showed the higher OD value of ALP activity (Figures 6b).

**Osteopontin and Osteocalcin Gene Expression**

**Figure 7:**
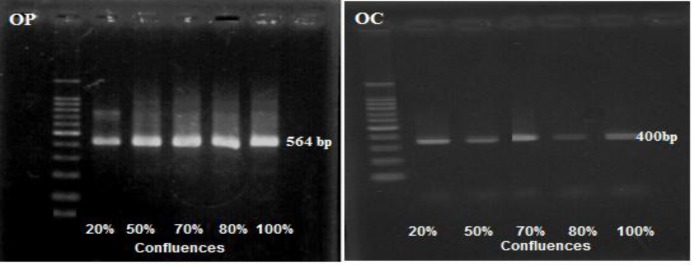
RT-PCR analysis of osteopontin (OP) and osteocalcin (OC) expression with gene specific primers on cells of 20, 50, 70, 80 and 100% confluent BMMSCs.

The effect of confluence on osteogenic gene expression was measured by RT-PCR analysis of osteopontin and osteocalcin expression with gene specific primers on cells of different confluences. It was found that BMMSCs-derived osteogenic cells expressed prominent osteopontin gene among all confluences. In contrast, osteocalcin gene expression was abundant in 70% confluent cells. In addition, 100% confluent cells showed abundant ostecalcin expression comparing to 20, 50 and 80% confluences (Figure7).

## Discussion

 In the field of cellular therapy, the degree of confluence to passage or harvest BMSCs remains an important silent factor. Therefore, the effects of confluence on properties of BMSCs were studied and confluence-associated osteogenic differentiation efficiency was identified. This study reflects the impact of cell density and microenvironment created by the increasing cell to cell contact on cell viability, CFU-F, population doubling, Brd-U incorporation, expression of ERK and P-ERK proteins and glucose consumption rate. Also, osteogenic differentiation efficiency was identified by determining calcium deposition, alizarin red staining, ALP activity and osteopontin and osteocalcin gene expression during culture expansion under conditions approved for clinical use. 

The cell density of expansion with different confluences was as expected. It was noticed that the cell density of expansion with different confluences was as expected. It was noticed that the highest cell density appeared when the cell achieved 100% confluence and the lowest one appeared at 20% confluence. At 70% and 80% confluence, density was significantly lower than that at 100% confluence and higher than 20% confluence. Therefore, there was a correlation between confluences % and the cell density. BMMSCS viability was declined at lower and higher confluences and there was no significant difference in viability between 20% and 100% confluence. Previous studies showed that there was no identical method for culturing MSC and standards for degree of confluence, cell densities and duration of cell expansion. Some studies proposed that culturing cells at low density resulted in more rapid proliferation.^41^^,^^42^ Others have noticed that keeping of very low cell densities through expansion was required to obtain homogeneous cell cultures with high proliferation and differentiation potential.^43^^,^^44^ Ren et al.^4^ reported that growing BMMSCs to higher confluences was associated with increased cell density and yield, but this high degree was detrimental to MSC that impaired self-renewal and differentiation capacity, supporting the hypothesis that cell-to-cell contact is harmful to MSC quality. BMSCs grown to 50% confluence also differ from 80% confluent cells, but these differences were less certain. On that way,Song et al.^45^showed that total number of cells and total cell density increased with time, but after that, the higher density cultures expanded more slowly accompanied with high apoptosis levels. 

Concerning CFU-F, the results obtained from this study revealed that as the confluence of BMMSCs increases until reaching 80%, the cells become highly able to form CFU-F, followed by decrease. These data showed that confluence affected BMSC colony formation, particularly for 100% confluent cells. The quantity of colonies formed by BMSCs was similar for 50% and 80% confluent cells but was less for 100% confluent cells. In addition, these data are in agreement to some extent with other works ^46^in which the dense colonies at low plating density and cell growth were likely to be inhibited at the colony center because of contact inhibition. At intermediate density, the growth pattern was a mix of colonies. Gregory et al.^47^suggested that avoiding overcrowding at the colony center might affect proliferation directly or indirectly through the induction of early differentiation of MSC.

The proliferation rate of BMMSCs at different confluences was assessed by Brd-U incorporation for 24 hours. The Brd-U uptake rate was significantly elevated as the confluence increased until reaching 80% confluence, followed by a decrease at 100% confluence. The massive inhibition of proliferation rate with high confluences obtained in this work was in agreement with previous studies in which^48^ BMMSCs plated at lower densities had a faster proliferation. A similar relationship was also found when the effect of seeding density on scaffolds was investigated.^49^ It was reported that the highest seeding density of cells was resulted in a slight increase in cell number compared to the lowest seeding density, which had a large increase in cell number. In addition, Colter et al.^41^reported that extremely low densities (0.5–12 cells/cm^2^) showed the size of single-cell-derived colonies that represent cell number and hence proliferation rate, to be inversely proportional to seeding density^50^.

The results of the population doubling showed that 70% and 80% confluences exhibit a significant increase in PDs when compared to that at 50% and 100% confluences. In most confluent BMMSCs, cell growth arrested in passage 12–13, whereas 70% and 80% confluent proliferation stopped in passage 14–16 In another study^51^, BMMSCs seeded at 4 different densities showed the results which were consistent with the current findings. They showed that seeding at lower density results in faster proliferation rate and PDs than those of a higher density. They also showed that cell characterization was not affected by seeding density as all cells had the same cell surface marker profiles. Another study explored the optimum seeding density for MSCs derived from different sources.^52^ Seeding density as a factor beside age of donor and gender affected proliferation rate and expansion of MSCs for clinical application. It was found that MSCs from a range of sources have faster proliferation rate/PDs at lower seeding densities.^50^PDT decreased by increasing confluence from 50% to 80% and then increased at 100% confluent.^4^

The confluence-associated proliferation was provided by measuring bands of ERK and its phosphorylation (P-ERK) by Western blot. Bands of ERK were expressed among all confluences of BMMSCs while bands of P-ERK were highly detectable in 70% and 80% confluent BMMSCs comparing to other confluences. Densitometric measures of band intensities found that ERK band intensity of 100% confluent BMMSCs was lower compared to other confluences. The intensities of remaining confluences bands were nearly the same, While the higher P-ERK band intensity was found in 70% and 80% confluent BMMSCs. P-ERK in 80% confluent BMMSCs was much higher than 20% and 50% confluent cells but slightly higher than 70% and 100% confluent cells. P-ERK in 70% and 100% was higher than 20% and 50% confluences. These data are parallel to other data identifying the cell proliferation and differentiation. Activated ERK phosphorylates cellular substrates, many of which are kinases whose activity prolong and proliferate the signaling cascade.^22^ Also, phosphorylation might play a pivotal role in the regulation of cell proliferation and differentiation.^20^

To identify signs other than microscopically identification of BMSC confluences, levels of glucose uptake by cells were measured in culture supernatants. All BMMSCs at all confluences are regular in glucose consumption after day 2. However, on days 3, 4 and 6, glucose consumption at 70% and 80% confluence was elevated. On the day 5 the consumption rates at confluence 50, 70, and 80% were higher than the lowest (20%) and highest (100%) confluences. On the basis of these data, we concluded that the value of glucose is a good indicator of cell number and can be used to determine the timing of BMSC passage or harvest as previously mentioned.^4^

Finding the optimum confluences for maximal osteogenic differentiation efficiency is useful in potential clinical applications. Calculation of the total area of Alizarin Red staining showed that BMMSCs had a very low area of staining at 20% and 50% confluence which indicated deficiency of in vitro osteogenic differentiation potential. 80% and 100% confluent BMMSCs showed higher staining than other confluences. Measurement of calcium deposition resulted in a more significant increase in BMMSCs cultured to 80% confluence than both 70% and 100% confluence. BMMSCs cultured to 100% confluence recorded more increases in the activity of ALP than other confluences. Expression of osteopontin gene was found among all confluences. In contrast, osteocalcin gene expression was more abundant in 70% and 100% confluent cells than others. Collectively, most markers of osteogenic differentiation are increased with the increasing % confluence even at 100%. These data are in the agreement with those of other investigators who worked in heterogeneous MSC populations^46^ or influence MSC phenotype during expansion.^53^^,^^54^^,^^55^

 On the contrary, seeding density has been shown to impact the efficiency of in vitro adipogenesis^55^ chondrogenesis^56^^,^^57^osteogenesis^58^^,^^59^ or myofibroblastogenesis^60^. They recorded that poor differentiation of various types of MSCs at high seeding density could be due to mechanical factors^42^^,^^59^^,^^60^. It was previously demonstrated that in vivo bone formation ability of 100% confluent BMSCs was reduced compared with 70% confluent BMSCs, but this difference was donor dependent.^61^^,^^62^It was concluded that the low density cultures give valuable effects, while high confluence cultures were resulted in reduced osteogenic and adipogenic differentiation.^61^Ren et al.^4^ showed that harvesting cells at 80% confluence was optimal because most bone biomarkers of gene expression was changed in 100% confluent cells suggesting that the genes that were up regulated in 100% confluent BMSCs were inhibitor genes and angiogenesis inhibitors. 

 Finally, it was concluded that 80% confluence is the best for proliferation or osteogenic differentiation. Previous studies collectively attributed it to the contact inhibition. The effect of confluence could possibly be different when BMMSCs are grown under other conditions or isolation of MSCs from other tissues. For example, it is not known if the effect of confluence would be the same as BMMSCs grown in bioreactors or scaffolds in combination with other media such as serum-free culture media or on other types or sources of stem cells. In addition, the effects of confluence on the in vivo properties of BMMSCs could differ from the properties measured in vitro. Unfortunately, none of these literatures discussed about the time needed for cells to take the amount of nutrition from media to find differentiation ability. Further investigations are required to answer the following questions: What is the exact confluence of cells to gain the normal shape tolerable for osteogenic differentiation? What is the exact confluent cell to communicate with other cells? What is the exact confluence to gain activating gene and surface receptors of cytokine and growth factors that are capable of work differentiation? 

## CONCLUSION

 Finally, it was concluded that 80% confluence is the best for proliferation or osteogenic differentiation. Previous studies collectively attributed it to the contact inhibition. The effect of confluence could possibly be different when BMMSCs are grown under other conditions or isolation of MSCs from other tissues. For example, it is not known if the effect of confluence would be the same as BMMSCs grown in bioreactors or scaffolds in combination with other media such as serum-free culture media or on other types or sources of stem cells. In addition, the effects of confluence on the in vivo properties of BMMSCs could differ from the properties measured in vitro. Unfortunately, none of these literatures discussed about the time needed for cells to take the amount of nutrition from media to find differentiation ability. Further investigations are required to answer the following questions: What is the exact confluence of cells to gain the normal shape tolerable for osteogenic differentiation? What is the exact confluent cell to communicate with other cells? What is the exact confluence to gain activating gene and surface receptors of cytokine and growth factors that are capable of work differentiation?
